# Effectiveness of an Intervention for Children with Externalizing Behavior and Mild to Borderline Intellectual Disabilities: A Randomized Trial

**DOI:** 10.1007/s10608-016-9815-8

**Published:** 2016-11-01

**Authors:** Hilde Schuiringa, Maroesjka van Nieuwenhuijzen, Bram Orobio de Castro, John E. Lochman, Walter Matthys

**Affiliations:** 1 Department of Developmental Psychology, Utrecht University, P.O. Box 80140, 3508 TC Utrecht, The Netherlands; 2 Department of Clinical Child and Family Studies, VU University Amsterdam, Van der Boechorststraat 1, 1081 BT Amsterdam, The Netherlands; 3 Learn Research Institute for Learning and Education, VU University Amsterdam, Amsterdam, The Netherlands; 4 The EMGO+ Institute for Health and Care Research, VU University Amsterdam, Amsterdam, The Netherlands; 5 Department of Clinical Psychology, University of Alabama, Tuscaloosa, AL USA; 6 The Conduct Disorder Prevention Research Group, University of Alabama, Tuscaloosa, AL USA; 7 Department of Child and Adolescent Studies, Utrecht University, Utrecht, The Netherlands; 8 Department of Psychiatry of University Medical Center Utrecht, Utrecht University, Utrecht, The Netherlands

**Keywords:** Externalizing behavior, Parent management training, Cognitive behavioral therapy, Mild to borderline intellectual disabilities

## Abstract

This study evaluated the effectiveness of Standing Strong Together (SST), a combined group based parent and child intervention for externalizing behavior in 9–16 year-old children with mild to borderline intellectual disabilities (MBID). Children with externalizing behavior and MBID (IQ from 55 to 85) (*N* = 169) were cluster randomly assigned to SST combined with care as usual or to care as usual only. SST led to a significant benefit on teacher reported but not on parent reported externalizing behavior. SST had significant effects on parent rated positive parenting and the parent–child relationship. The present study shows that a multicomponent group based intervention for children with MBID is feasible and has the potential to reduce children’s externalizing behavior and improve both parenting behavior and the parent–child relationship.

## Introduction

Research on the effectiveness of interventions in children with externalizing behavior problems and average intelligence is extensive. According to a meta-analysis in children and adolescents (McCart et al. 2006), the mean effect size (ES) of behavioral parent training (0.47) and of cognitive behavioral therapy (0.35) are in the small to medium range (Cohen 1988). In addition, studies have shown that the combination of behavioral parent training and cognitive-behavioral child therapy provides more improvements than parent-focused or child-focused intervention alone (Kazdin et al. 1992; Lochman and Wells 2004; Webster-Stratton and Hammond 1997).

However, it is unclear whether combined parent training with child cognitive behavioral therapy is equally beneficial to children with mild to borderline intellectual disabilities (MBID; IQ 55–85). This is unfortunate, as children with MBID, including children with mild intellectual disabilities (IQ 55–70) and borderline intelligence (IQ 70–85), have a three to four times higher risk of developing externalizing behavior problems compared to their peers with average intelligence (Baker et al. 2002; Dekker et al. 2002) and children with MBID and externalizing behavior problems have been found to be overrepresented in child welfare and justice systems (e.g., in the Netherlands; Kaal 2010). Moreover, these children’s externalizing behavior problems tend to be more persistent over time than in children with average intelligence (defined here as an IQ above 85) (Emerson et al. 2011; Green et al. 2005). The development of intervention programs for these children therefore is important both for the treatment and the prevention of externalizing behavior problems (Einfeld et al. 2011).

Surprisingly, though, only few intervention studies targeted children with MBID and externalizing behavior problems, and most of these studies examined the preventive effects of programs targeting parents of preschool children, without a child intervention component (Hand et al. 2012; McIntyre 2008a, b; Plant and Sanders 2006; Roberts et al. 2006; Tellegen and Sanders 2013). Concerning parenting programs, results of the first studies targeting parents of children with MBID and externalizing behavior problems are promising (Matson et al. 2009), but the studies are limited in terms of their design (pre-post, no control condition), sample size (typically less than 25 and always less than the minimally recommended 35 per condition), and lack of differentiation in participants’ level of intelligence (for a review see Einfeld et al. 2013). These limitations may be understandable due to the intricacies of recruiting, randomizing, and participating in randomized trials for these families, but a rigorous larger scale randomized trial is needed to establish whether the promising effects suggested by these studies are actually attained in daily practice.

Concerning cognitive behavior therapy with clients with MBID and externalizing problems themselves, the only randomized trials of cognitive behavioral therapy for externalizing behavior with people with MBID we know of have been conducted with adults. A group based cognitive behavioral intervention proved to be effective in improving anger control in adults with MBID (Willner et al. 2013). Thus, well-designed randomized and sufficiently powered trials of the effects of multimodal or cognitive behavioral training with children are dearly needed.

In theory, it is plausible that mild to borderline intellectual disabilities complicate the use of cognitive demanding cognitive techniques (like cognitive restructuring, challenging thoughts and beliefs, mentally compering multiple expected outcomes of behaviors) with children. Children with MBID show deficits in cognitive skills that are important for such cognitive techniques (Sturmey 2004), such as sustained attention, working memory, verbalizing feelings, and distinguishing between thoughts, feelings, and behavior (Joyce et al. 2006; Van Nieuwenhuijzen et al. 2009). In the literature and in clinical practice, it has been assumed that children with MBID would benefit less from cognitive behavioral treatment due to their lower cognitive functioning (e.g., Sturmey 2004).

However, it might alternatively be that cognitive behavioral therapy can be effectively conducted with children with MBID, provided that the intervention program is specifically adapted to the cognitive abilities of children with MBID and their parents. Pioneers in cognitive behavioral therapy with children have argued that evidence-based cognitive techniques can work in clients with complex co-morbidities, provided that adaptations are made to accommodate client characteristics (Kazdin and Whitley 2006). This hypothesis is in line with developmental research in a number of domains that showed young children to be much more cognitively advanced than was assumed, if only demand and task characteristics were adapted to their verbal development and attention span (for example concerning Theory of Mind; Wellman et al. 2001). Given the divergence in opinions about the usefulness and feasibility of cognitive intervention techniques with children with MBID, empirical data on the actual applicability of these techniques with these children is needed. We therefore aimed to test whether cognitive behavioral treatment can be used effectively by children with MBID when the cognitive assignments are adapted to their cognitive capabilities with regard to language, attention span, working memory, and need to rehearse.

The aim of the present study is to examine the effectiveness of the multicomponent intervention program Standing Strong Together (SST), that combines parent-management training and cognitive behavior therapy to reduce externalizing behavior problems in children with MBID. SST is an adaptation of the Utrecht Coping Power Program (Van de Wiel et al. 2007; Zonnevylle-Bender et al. 2007), an abbreviated version of the Coping Power Program (Lochman et al. 2008; Wells et al. 2008). The Coping Power Program and the Utrecht Coping Power Program were found to be effective in improving behavior and reducing rates of delinquency and substance use in aggressive boys and in children with disruptive behavior disorders (Lochman and Wells 2002, 2004; Van de Wiel et al. 2007; Zonnevylle-Bender et al. 2007). For treatment purposes, a multicomponent program consisting of a parent training intervention and a cognitive behavioral intervention seems appropriate. Indeed, specific parenting characteristics have been found to play a role in the development and persistence of externalizing behavior problems both in children with average intelligence and MBID (Dodge and Pettit 2003; Hoeve et al. 2009; Lansford et al. 2004; Schuiringa et al. 2015). For example, harsh and inconsistent parenting has been found to be a predictor of the persistence of conduct problems in children with an IQ below 70 (Emerson et al. 2011). Furthermore, parents of children with MBID often perceive higher levels of parenting stress, compared to parents of children with an average intelligence (Hastings 2002; Hastings and Beck 2004) and more often state that help is needed (Douma et al. 2006).

A second important factor in children’s externalizing behavior problems is impaired social information processing, as has been found in samples of children with average intelligence (Crick and Dodge 1994; Matthys et al. 1999) and MBID (Van Nieuwenhuijzen et al. 2005, 2006b). For example, children with MBID and externalizing behavioral problems generate more aggressive responses to hypothetical conflict vignettes than children with MBID but without behavior problems (Van Nieuwenhuijzen et al. 2005). Thus, the child’s social cognitions may be targets for interventions aiming to reduce externalizing problem behavior.

We conducted a relatively large-scale cluster-randomized multi-informant trial to provide a robust test of the effectiveness of combined parent management training and cognitive behavior therapy in families of children with MBID and externalizing behavior problems. An add-on design was used comparing SST combined with care as usual (CAU) to CAU only. We tested the hypotheses that SST combined with CAU compared to CAU alone would be: (1) effective in reducing externalizing behavior problems and aggressive social cognitions in children with MBID, and (2) effective in improving parenting behavior and the parent–child relationship. In addition, we tested the hypothesis that (3) the effect of SST would be larger in older children and in children with a higher IQ.

## Method

### Design

This multicenter, parallel group, cluster randomized controlled trial was conducted in the Netherlands. Twelve centers for the treatment of children with MBID and externalizing behavior, geographically distributed across the country, participated. All participating treatment centers offered care at two locations at least. The intervention was randomized at the location level, stratified by treatment center. Two different locations of each treatment center were randomly allocated to the intervention or control condition, by flipping a coin (randomization ratio 1:1). We ensured that the intervention group was physically separated from the control group to prevent contamination from the intervention to the control condition. Randomization at the location level also made treatment center characteristics, such as CAU, and demographic characteristics (as a result of the region in the Netherlands) similar in both conditions. Families receiving treatment at the participating locations and meeting the inclusion criteria were asked to participate. At each location, three to five families were selected to participate in the study. Thus, the number of participants per location and condition is not exactly equal. The pre-test was conducted immediately prior to the beginning of the intervention, and the post-test immediately after the intervention period. The study was conducted in three consecutive years (2009, 2010, and 2011). The Medical Ethical Committee of the participating university approved the study (CCMO nr 08/249).

### Procedure

First, children were selected to participate in the present study when (1) they scored above the 90th percentile on one or both of two subscales (Aggression and Rule Breaking) of the Child Behavior Check List (CBCL, see Measures) either reported by their parent(s) or the care staff, (2) they were living at home with their parents or caregivers (adoption parents or a biological parent and stepparent), (3) children and their parents were able to communicate in Dutch, and (4) provided consent. Any children or parents suffering from active psychosis, severe vision problems, or severe hearing problems were excluded from the study. In addition, children with a clinical diagnosis of autism spectrum disorders were excluded from the study, because the intervention was not designed to meet their specific needs.

Second, consent was obtained from the parents when treatment centers sent out the letters with detailed information about the study and a request for written consent. Researchers and care staff provided additional information when needed. The parents completed questionnaires during a home visit. The researcher used a list with synonyms and explanations of difficult words to ensure clear and unambiguous responses to participants’ questions about the questionnaires. The researcher posed the questions and recorded the answers on the form. In the week following the home visit a short questionnaire was administered during two phone calls. The child measures were individually administered by a research assistant from the university in a separate and quiet room at the child’s school. Child assessments were performed only during the first two years of data collection, due to feasibility reasons. The teachers completed a questionnaire about the child’s behavior that was sent and returned by mail. Children received a small gift for their participation. Parents received a gift token (€10 for each assessment).

### Participants

One hundred sixty-nine families with a child with MBID aged 9–16 years participated in the study. The participating children were receiving treatment for their accompanying externalizing behavior problems in day care and outpatient treatment centers in the Netherlands. Children were living at home with their biological parents or other legal caregivers (adoption parents or a biological parent and stepparent). The children received treatment either in outpatient or day-care treatment, both in the intervention and control condition. Twelve out of 21 Dutch special treatment centers participated in this study. The participating treatment centers offered care at least at two locations. All these treatment centers require (1) the children to have an IQ in the range of 55–85 and (2) the children to demonstrate severe adjustment problems in one or more social contexts as well as impairments in their daily functioning, due to their intellectual disability and accompanying externalizing behavioral problems.

We are aware that our definition of intellectual disabilities differs from that most often used for mild intellectual disabilities in the international literature (IQ 50/55–70). We adopted the broader definition of mild intellectual disabilities as used in the Netherlands (IQ 55–85). In the Dutch situation, individuals with borderline intelligence (71–85) with severe limitations in adaptive functioning are also included in the healthcare and special education system for individuals with mild intellectual disabilities. Children with mild intellectual disabilities (MID) and children with borderline intelligence (BID) with severe limitations in adaptive functioning are present in both of the settings from which we selected participants for the intervention and control group. Moreover, the children with MID (IQ 55–70, 37 % of the sample) did not differ significantly from the children with BID (IQ 71–85, 63 % of the sample) on social information processing, parenting and externalizing behavior problems, in the present study.

**Fig. 1:**
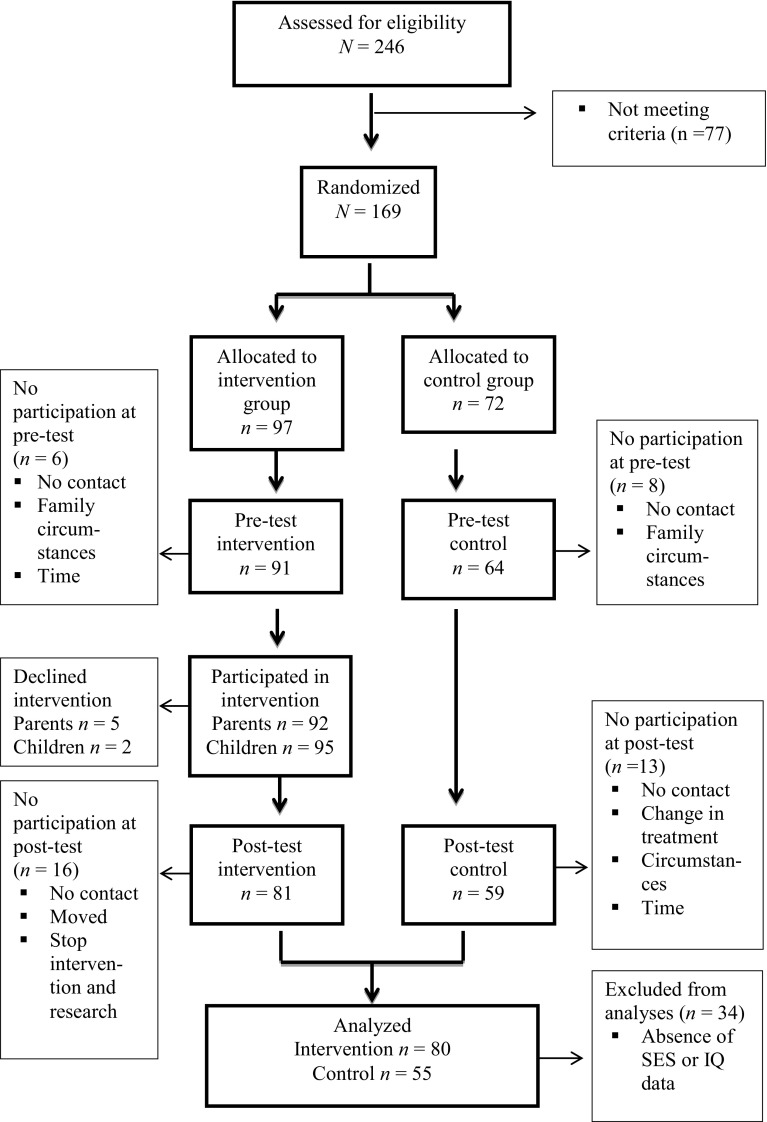
Flow chart of randomization design

Participant flow of parents is shown in Fig. 1. Clinical workers were requested to make a pre-selection of suitable families based on our inclusion criteria and asked these parents to participate. Two hundred forty-six families were invited and willing to participate and therefore assessed for eligibility. Seventy-seven children from these families did not score above the 90th percentile of the CBCL. Thus, 169 families signed informed consent. As locations were randomly assigned, the number of participants in each condition depended on location. Thus, 97 participants were included in the intervention group and 72 in the control group.

Table 1 lists demographic characteristics for the intervention and control group. The mean age of the participating children was 12.5 years (*SD* = 1.99) and children had a mean intelligence score of 74.2 (*SD* = 10.44). When possible, both parents participated in the study and jointly completed one set of questionnaires about their child’s behavior, parenting, and the parent–child relationship. When this was not possible, the main caregiver was asked to complete the questionnaires. The majority of the sample was of a Dutch origin (81.8 % of the mothers, 71.7 % of the fathers). A minority of the sample was of an Antillean origin (5.8 % mothers, 5.3 % fathers), Moroccan background (3.9 % mothers, 5.9 % fathers), Surinamese background (1.9 % mothers, 5.9 % fathers), or Turkish background (1.9 % mothers, 4.6 % fathers). 4.5 Percent of the mothers and 6.6 % of the fathers scored ‘other’. The ethnicity for this remaining group was not further specified. The participating families had a mean SES of 4.4, indicating a parental educational level of lower vocational education. As there was not enough variance in SES data, SES was not used as a moderator in the analyses.

**Table 1:** Sample characteristics and group differences by condition at pre-test

	Intervention group *M (SD)*	Control group *M (SD)*	*F*	*p*
Child				
Gender (% boys)	75	66,7	*χ* ^*2*^ = 1.5	.22
Age (years)	12.34 (2.05)	12.80 (1.89)	2.01	.16
IQ	74.82 (10.63)	74.45 (10.21)	0.61	.44
SES (1–10)	4.52 (2.10)	4.13 (1.96)	1.43	.23
TRF T-score	66.87 (10.02)	66.92 (9.54)	0.04*	.84
CBCL T-score	67.54 (7.12)	67.39 (7.78)	0.14*	.71

*SES* Social economic status, *TRF* teacher report form externalizing behavior, *CBCL* child behavior checklist externalizing behavior

* Raw scores were used to test for differences between groups

Sixty-eight percent of the questionnaires were filled out by mothers, 12 % by fathers and in 20 % both parents filled out the questionnaire together. Ninety-two percent of the children, who filled out the questionnaires at pre-test, also filled out the questionnaires at post-test. Teachers did not complete the questionnaire for 18 % of the children at pre-test, and for 24 % of the children at post-test. Parent reports were missing completely at random on both pre (8 % missing)- and post-test (17 % missing) (pre-test: Little’s MCAR test: X^2^/4 = 0.60, *p* = .66; post-test: Little’s MCAR test: X^2^/4 = 0.41, *p* = .80). Therefore, parent reported data was imputed at pre- and post-test. Multiple imputation is currently recommended as modern missing data handling technique (Baraldi and Enders 2010). We used Multiple Imputation techniques module of LISREL 8.7 with the Expected Maximization (EM) algorithm. Teacher reports were not missing at random at pre-test (pre-test: Little’s MCAR test: X^2^/4 = 5.49, *p* = .00; post-test: Little’s MCAR test: X^2^/4 = 0.99, *p* = .41), therefore teacher data was not imputed at pre- and post-test. Data collection on social information processing in children was no longer feasible during the last year of data collection; therefore, children’s missing social information processing data was not imputed.

### The Intervention

SST is a manualized behavioral parent–child intervention based on the evidence-based Utrecht Coping Power Program (Van de Wiel et al. 2007; Zonnevylle-Bender et al. 2007). SST consists of a group-based parent intervention combined with a parallel group-based social skills and social problem solving intervention for children. Children with MBID have special needs concerning treatment (Van Nieuwenhuijzen et al. 2006a). In line with recommendations made in the literature (De Wit et al. 2011) adaptations that were made to the Utrecht Coping Power Program included: (1) additional psycho-education for parents concerning children with MBID, (2) more repetition of topics throughout the intervention, (3) more visual cues, (4) less information per session, (5) an active approach with less passive instructions and more activities and exercises, and (6) simplified language (De Wit et al. 2011). The parent intervention empowered parents with a collaborative approach as used in the Incredible Years intervention (Webster-Stratton 2001). For use in families of children with MBID, adaptations were made with help of experienced clinical workers.

The child intervention was conducted in a group with a maximum of five children in twelve 75-min weekly child sessions. The minimum number of participants in an intervention group was three, in order to ensure group elements, exchanging between participants, and exercise possibilities. The parents of the children in a child intervention group participated in a parallel parent group, consisting of ten 90-min parent sessions, every other week. The themes in the parent and child intervention overlapped as much as possible. The children received intervention during school or day care hours at the day care treatment center. The dates for the parent sessions were set in collaboration with the participating families at the day care treatment centers. Travel costs were reimbursed.

The parent intervention is primarily focused on improving parenting skills that affect the parent–child interaction. First, parents learn how to create the conditions for a child to listen and follow instruction. Parents practice with setting rules and giving instructions. Several sessions then focus on the use of praise and tokens. Later sessions focus on ignoring, the use of time-out, and loss of privileges. Finally, parents learn how to ask for help and taking care of themselves. Sessions start with a retrospect to the previous session and reviewing the parents’ experience with the skills covered the previous week. New skills are then practiced using role-play, feedback, modeling and exchange between parents. Sessions end with discussing the home assignment and summary of the children’s session. The following theme’s are covered in the parent intervention: (1) acquaintance and psycho-education, (2) structure and rules, (3) instructions, (4) praise, (5) use of reward, (6) ignoring, (7) boundaries and time out, (8) loss of privileges, (9) helping thoughts, (10) support.

In the child intervention, several sessions concentrate on recognizing feelings, in particular feelings of anger. Children learn to identify cognitive or physical indicators of emotional reactions to provocation or frustration. Using an anger thermometer children learn to recognize different levels of their anger. The recognition of these emotions eventually leads to focus on methods for anger management. In addition, children learn social problem solving skills: they learn to define problems that they encounter, accurately understand another person’s intention, generate solutions and choosing an appropriate solution. A metaphor of three soccer players is used; the angry, scared and assertive soccer player. The characters of these soccer players help children to discover appropriate and less appropriate solutions. Later sessions address issues related to peer pressure. All sessions start with a retrospect to the previous session and reviewing the children’s’ experience with the skills covered the previous week. The skills are practiced using for example role-play, videotapes and memory games. Sessions end with discussing the home assignment for the next week.

The following theme’s are covered in the child intervention: (1) acquaintance, (2) communication, (3) everyone is unique, (4) helping thoughts, (5) recognizing different emotions, (6) feelings of anger, (7) handling various degrees of anger, (8) social problem solving, (9) handling bullying, (10) collaborate with other children, (11) handling peer pressure, (12) ending and summarizing.

### Clinical Staff Training

Clinicians at the treatment centers provided both routine care and the SST intervention. A social worker and a group leader provided the parent intervention. The same group leader and a therapist provided the child intervention. To become certified as a SST trainer, clinical staff had to attend a one-day workshop and observe and participate in three out of four supervision meetings (after every three child sessions), directed by the developers of the intervention. The workshop and supervision meetings were attended by all clinicians providing SST and supervised by accredited SST trainers, to ensure that the program was delivered with fidelity and to provide the opportunity to discuss problems encountered while providing SST.

Clinical staff providing SST received a one-day training course guided by the developers of the intervention program. The training course consisted of an introduction with information about the theoretical background of the program and practical tips with regard to the implementation of the intervention. In the afternoon session, future trainers practiced their trainer skills by participating in and reflecting on role-plays handling topics such as motivating parents to join in a role-play, creating a safe atmosphere during the first session, distribution of attention for all participants, increasing desirable behavior and decreasing undesirable behavior, and explaining exercises from the protocol. During these 3 h supervision sessions, trainers bring in topics they would like to discuss, practice, or reflect upon with other trainers.

Teachers and day care group leaders were closely involved in the intervention. The clinical staff conducted an information session for the teachers and involved day care leaders before the start of the intervention. In addition, after each session teachers and care group leaders received a summary of session content. Parents received a summary after every child session.

Families in the intervention condition received SST in addition to CAU. The children in the control condition received CAU alone within their treatment center. Treatment for children with MBID and externalizing behavior problems is eclectic, including a wide range in intensity and content between treatment centers. However, for a broad majority of the participants CAU consisted of a combination of child behavior management in daycare treatment, parental guidance, and additional individual treatment for children (e.g., social skills training, creative therapy, psychomotor therapy, drama therapy). In contrast to SST, CAU parent support was not manualized. For children, SST was more cognitive behaviorally oriented and more structured than CAU, as hardly any manualized interventions were used in CAU.

### Measures

#### Child Behavior Checklist and Teacher Report Form

Parents, teachers, and care staff completed the Dutch version of the Externalizing Behavior subscale of the Child Behavior Checklist (CBCL, Achenbach and Rescorla 2001; for the Dutch version see Verhulst et al. 1996) or Teacher’s Report Form (TRF, Achenbach and Rescorla 2001; for the Dutch version see Verhulst et al. 1997). Reliability was high in this study; Cronbach’s alphas were ≥.89. In the analyses, raw scores on the Externalizing Behavior scale were used. Using Dutch norms, T-scores on the Externalizing Behavior subscale were calculated for descriptive purposes only.

#### Parent Daily Report

Additional behavioral ratings by parents were obtained with the Parent Daily Report (PDR; Chamberlain and Reid 1987). The parent was asked to indicate occurrence or nonoccurrence in the previous three days of the behavior mentioned in the items. A time period of three days, instead of 24 h, was chosen as participants spent a substantial part of their time away from home at school and day care, where they were treated for their behavioral problems. Cronbach’s Alpha was .92 at pre-test, and .93 at post-test.

#### Alabama Parenting Questionnaire and Ghent Parental Behavior Scale

We used five scales from the Dutch translation of the Alabama Parenting Questionnaire (APQ; Shelton et al. 1996) to measure parenting characteristics, including Parental Involvement, Positive Parenting, (poor) Monitoring, Physical Punishment, and Positive Discipline. As the APQ does not include a subscale on rule setting, we added this subscale of the Ghent Parental Behavior Scale (GPBS; Van Leeuwen and Vermulst 2004). Items of the GPBS Physical Punishment scale were also used to combine with several items of the APQ Harsh Punishment scale. A number of studies (e.g., Shelton et al. 1996) provided support for adequate reliability and validity of the APQ. The Parental Involvement, Positive Parenting, Monitoring, Positive Discipline, and Rule Setting subscales were combined into a Positive Parenting scale to be able to include the wide variety of items and to also minimize the number of outcome variables used in the analyses. Negative Parenting includes the combined Harsh Punishment and Physical Punishment scales. Both composite scales had moderate to a high Cronbach’s alpha in the current study; Positive Parenting .81 at both pre- and post-test, and Negative Parenting .75 at pre-test and .68 at post-test.

The Negative Parenting scale was not normally distributed after log transformation was performed. However, in this study the N is large enough to assume normal distribution of the data and therefore to use the original analyses (Hays 1973). The Negative Parenting scale was used both continuous and dichotomized (0 = *no use of negative parenting*, 1 = *use of negative parenting*) in the effectiveness analyses.

#### Parenting Stress Index

Parents completed the Dutch version of the Parenting Stress Index (PSI; Abidin 1983; De Brock et al. 1992) to assess the parent–child relationship. The subscales Acceptance, Sense of Competence, and Attachment were combined into a parent–child relationship scale. The reliability and validity of the Dutch version of the PSI are sufficient (De Brock et al. 1992). Cronbach’s alphas were high in the current study: .84 at pre-test and .90 at post-test.

#### Wechsler Intelligence Scale

An estimate of the intelligence of the participants was obtained using the Vocabulary and Block Design subtests from the Dutch version of the Wechsler Intelligence Scale (WISC-III, Kort et al. 2005, Silverstein 1970b). These two subtests, taken together, have been shown to correlate more strongly (*r* = .86) with the complete WISC-III than any other subscale, and thus provide an accurate estimate of children’s overall intelligence (Silverstein 1970a). The same WISC-III subtests have also been used in previous research to estimate the intelligence of children with MBID (e.g., Van Nieuwenhuijzen and Vriens 2012).

#### Social Problem Solving Test and Normative Beliefs About Aggression Scale

Aggressive social cognitions were assessed with a combination of the Social Problem Solving Test revised for children with MBID (SPT-MID; Van Nieuwenhuijzen et al. 2001) and the Normative Beliefs About Aggression Scale (NOBAGS; Huesman and Guerra 1997). The Social Problem Solving Test asks for social problem solving strategies in response to five hypothetical situations on video-tape. The SPT is described extensively in the Van Nieuwenhuijzen et al. (2006b) study. The SPT was administered and (double) scored by trained graduate students and a research assistant.

Children’s normative beliefs about aggression were assessed with the Normative Beliefs About Aggression Scale (NOBAGS; Huesman and Guerra 1997). Strong support for the reliability and validity of the NOBAGS has been provided (Huesman and Guerra 1997), including children with MBID (Van Nieuwenhuijzen et al. 2006b). For the present study, the NOBAGS total scale was combined with the SPT’s Aggressive Response Generation and Aggressive Response Decision subscales. Scores on the three subscales were standardized and summed. This aggregated scale was highly reliable, pre-test α = .90 and post-test α = .87.

### Treatment Integrity

As treatment integrity affects the effectiveness of intervention programs (Durlak and Dupre 2008), the intervention program needs to be delivered as originally intended. Clinical staff providing SST was selected based on their experience with providing group training to this target group, or because they were the regular therapists of children in the intervention group. All trainers attended the one-day SST workshop and supervision meetings to become a certified trainer.

To measure treatment integrity for research purposes, all intervention sessions in this study were audio taped. A random selection of 10 % of the sessions was scored on adherence, competence, and enthusiasm. Adherence was rated using a 3-point scale (totally, partly, not at all) on which the coder indicated to what level particular goals and practicing skills for each session were performed according to protocol. These items were adapted to the specific content of every session. In addition, three general items that were similar for every meeting were coded on adherence (e.g., ‘Discuss summary and new home assignment’). Competence was coded using a 3-point scale (Not at all, Sometimes, Very often) on 7 items (e.g., divide attention among group members, keep order, structure). The coder gave grades on Enthusiasm of trainers, ranging 1 = *Not at all enthusiastic* to 10 = *Very enthusiastic* on three items, such as ‘The trainer is enthusiastic’.

### Data Analyses

We tested whether the multilevel structure of the data required multilevel analyses in Hierarchical Linear Modeling (HLM). Variance between components was calculated for each of the outcome measures in order to check whether outcomes were associated with location. *No* significant amount of variation at the group level was found for any of the outcome measures, except for child behavior reported by parents on the PDR. Therefore, the design effect could be ignored and was not controlled for in the analyses (Muthén 2000). First, we tested for possible differences at baseline between the intervention and control group. Second, we examined intervention effects using a series of repeated measures ANCOVA’s. Gender, IQ, age, and SES were included as covariates as all of these variables were related to some of the outcome measures and it was theoretically plausible that these factors influenced the intervention effect. We wanted to prevent these factors from confounding the results. In addition, in a heterogeneous sample, controlling for confounding variables will reduce variance and therefore increase power. Third, to test for moderating effects of gender (0 = *girls*, 1 = *boys*), IQ (0 = *IQ* ≤ *75*, 1 = *IQ* > *75*), age (0 = 9–12, 1 = 13–16), and treatment center characteristics, we conducted additional repeated measure ANCOVA’s controlling for gender, IQ, age, and SES (except for the covariate that was included as a moderator), and interactions of the moderators with condition were tested. We additionally explored moderating effects of treatment center characteristics such as Type of Care (0 = *day care*, 1 = *outpatient treatment*), offered Help to Parents in CAU (0 = *no help*, 1 = *help*), and gender. Effect sizes were calculated as the standardized mean difference with mean gain scores. An effect size of .20 was considered small, .50 was considered medium, and an effect size of .80 was considered large (Cohen 1988). In addition, as secondary analyses, we examined whether the level of externalizing behavior of children declined from a clinical/subclinical level to the normal range for more intervention than control children. The percentage of children in the (sub)clinical range and normal range was calculated at pre and posttest and compared between intervention and control group with Chi-square tests.

## Results

The intervention and control group did not differ on baseline levels on any of the outcome variables or demographic characteristics gender, age, IQ, or SES.

### Treatment Integrity

Audio scoring indicated that all intervention sessions were completed. On average 75 % of all exercises was performed by the trainers. Trainers performed 85 % of the general parts of the sessions. Trainers were considered competent 72 % of time over all sessions. Trainers were rated as enthusiastic over 68 % of sessions. These numbers indicate that SST was by and large performed as intended (Durlak and Dupre 2008).

### Attendance

An average of seven out of ten sessions was attended by at least one of the parents. Some parents attended the intervention as couples (44 %), but the majority were mothers or fathers participating in the intervention alone (56 %). Sixty-nine percent of the parents participated at least seven out of ten sessions, and 24 % of parents attended all 10 sessions. The children attended an average of 11 out of 12 sessions, 83.7 % participated in at least ten out of twelve sessions. Most children participated in all child sessions (45 %), several attended no more than one session (3.5 %).

### Intention to Treat Intervention Effect

Results of the intention to treat analyses are presented in Table 2. The effectiveness of the intervention is tested with the interaction between treatment condition and time in each repeated measures ANCOVA. We found several significant intervention effects. First, a significant intervention effect on child behavior was found for teacher reported Externalizing Behavior (TRF). Teachers in the intervention group reported a decrease of externalizing behavior problems, while teachers in the control group reported an increase of externalizing behavior problems. No significant intervention effect was found for Externalizing Behavior on the CBCL and PDR.

**Table 2:** Means, standard deviations and repeated measures ANOVA results across all measures in intention to treat analyses controlled for SES, IQ, gender, and age

Measure	Intervention group *M (SD)*		Control group *M (SD)*		Time effect	Interaction effect	Cohen’s d
	Pre-test	Post-test	Pre-test	Post-test	*F(p)*	*F(p)*	
CBCL T-score	67.74 (7.00)	63.80 (7.65)	67.09 (8.13)	64.40 (9.17)	1.59 (.21)*	2.59 (.11)	0.19
PDR ext. behavior	0.34 (0.15)	0.29 (0.17)	0.29 (0.19)	0.25 (0.16)	0.45 (.51)	0.38 (.54)	0.06
TRF T-score	66.29 (9.35)	64.65 (9.09)	65.06 (10.08)	65.70 (8.29)	1.64 (.20)*	4.15 (.045)	0.25
Positive parenting	2.75 (0.38)	2.78 (0.38)	2.79 (0.41)	2.75 (0.35)	0.33 (.57)	4.51 (.04)	0.18
Negative parenting	0.27 (0.31)	0.16 (0.30)	0.25 (0.47)	0.16 (0.31)	0.42 (.52)	0.26 (.61)	0.06
Parent–child relationship	2.83 (0.31)	3.10 (0.49)	3.00 (0.60)	3.08 (0.75)	0.05 (.83)	5.74 (.02)	0.33
Aggressive social cognitions^a^	−0.08 (2.29)	0.42 (2.48)	0.42 (2.63)	−0.03 (2.22)	0.82 (.37)	7.73 (.01)	−0.40

*CBCL T*-*score* Child behavior checklist, subscale externalizing behavior, *TRF* teacher’s report form, subscale externalizing behavior, *PDR* parent daily report

^a^Standardizes scores

* Raw scores were used to test for time and interaction effects

Second, regarding parenting behavior, a significant intervention effect was found for Positive Parenting. Parents in the intervention group reported an increase on the use of positive parenting, while positive parenting according to parents in the control group decreased slightly. Also, a significant intervention effect was found for the parent–child relationship. As shown in Table 2, both parents in the intervention and control group reported an improvement of the parent–child relationship at post-test. This increase was significantly stronger in the intervention group. No significant intervention effect was found on Negative Parenting.

Third, for child social cognitions, there was a significant Time × Condition effect on Aggressive Social Cognitions. Children in the intervention group increased on aggressive social cognitions from pre- to post-test, while the control group decreased in using aggressive social cognitions. However, Aggressive Social Cognitions were not related to Externalizing Behavior (CBCL Externalizing Behavior *r* = .04, *p* = .73; TRF Externalizing Behavior *r* = −.11, *p* = .32; PDR *r* = −.11, *p* = .30).

### Moderator Analyses

IQ did not moderate the intervention effect. In addition, gender and age did not moderate intervention effects on any of the outcome variables. Moderating effects of treatment center characteristics such as Type of Care (day care treatment or outpatient treatment) and Help to Parents (treatment centers do or do not include parents in care as usual) were exploratory tested. A significant moderation effect was found on Aggressive Social Cognitions (*F*(1, 70) = 8.04, *p* = .006). SST had a positive effect for children treated in outpatient treatment, as their aggressive social cognitions reduced at post-test, compared to the control group. In contrast, SST had a negative effect on children receiving treatment in day care treatment centers, as their aggressive social cognitions increased at post-test compared to the control group. A separate post hoc analysis for the outpatient group only indeed indicated a trend effect towards a positive intervention effect (*F*(1, 6) = 3.18, *p* = .099). The other treatment center characteristics did not moderate intervention effects on any of the outcome variables.

As additional analyses, the drop from the (sub)clinical to the normal range was tested on both the CBCL and TRF T-scores. Children were selected by the institutions based on their score above the 90th percentile of aggression and/or rule breaking on the CBCL reported by the day care group leader or parents. During pre- and post-test parents and teachers reported on externalizing behavior. For the broadband scale Externalizing Problems, T-scores higher than 60 fall in the (sub)clinical range, and T-scores beyond 60 fall in the normal range.

At pretest, parents reported child externalizing behavior of the children in the control group in the (sub)clinical range for 87 % of the children, and in the normal range for 13 % of the children. In the intervention group, 89 % was rated as (sub)clinical and 11 % as normal. These differences between groups were not significant (*χ* ^*2*^ (*n* = 169) = .05, *p* = .82).

At posttest, for the control group, parents reported child externalizing behavior for 81 % in the (sub)clinical range, and 19 % in the normal range. In the intervention group, 75 % of the children was rated in the (sub)clinical range, and 25 % in the normal range. The differences in percentages between groups were, however, not significant (*χ* ^*2*^ (*n* = 169) = .67, *p* = .42), indicating that the intervention did not significantly increase recovery according to parents.

At pretest, teachers reported child externalizing behavior of the children in the control group in the (sub)clinical range for 79 % of the children, and in the normal range for 21 % of the children. In the intervention group, 71 % was rated as (sub)clinical and 29 % as normal. These differences between groups were not significant at pre-test (*χ* ^*2*^ (*n* = 138) = 1.05, *p* = .31). At posttest, for the control group, teachers reported child externalizing behavior for 80 % in the (sub)clinical range, and 20 % in the normal range. In the intervention group, 62 % of the children was rated in the (sub)clinical range, and 38 % in the normal range. The differences in percentages between groups were significant at posttest (*χ* ^*2*^ (*n* = 128) = 4.72, *p* = .03), indicating that the intervention did significantly increase recovery compared to the control group, according to teacher ratings.

## Discussion

The combined parent and child intervention Standing Strong Together for children with behavior problems and MBID modestly reduces teacher-reported externalizing behavior problems, increases positive parenting, and improves the parent–child relationship. Yet results of this first RCT in children with externalizing behavior problems and MBID are mixed in three ways. First, SST led to a significant benefit on teacher reported externalizing behavior problems, but not on parent reported externalizing behavior problems. Second, SST led to significant benefits on positive but not on negative parenting behavior. Third, contrary to expectations, SST led to an increase in aggressive social cognitions for the subgroup of children in day care, compared to a reduction of aggressive social cognitions in the control group, even though this increase in aggressive social cognitions was not related to changes in behavior, and limited to children in day care. No moderation by IQ was found, which means that no differences occurred in treatment effectiveness between children with an IQ ranging from 55–70 or 71 to 85.

It seems possible to change child externalizing behavior problems, parenting behavior and the quality of the parent–child relationship in children with MBID to some extent. Although intervention effects on child behavior and parenting were mixed from the perspective of statistical significance, effects were consistent in direction and modest range of magnitude. Indeed, effect sizes for externalizing behavior, parenting behavior, and the parent–child relationship measures were all in favor of the intervention condition. However, effect sizes were small. In particular, effect sizes of changes in child externalizing behavior problems were smaller than those in studies including children with average intelligence (McCart et al. 2006), possibly as a result of impaired social learning processes in children with MBID (Matthys et al. 2012). Nevertheless, these findings seem promising.

It should be noted that in the present study an add-on design was used, i.e., SST in combination with CAU was compared to CAU. A meta-analysis of evidence-based youth psychotherapies versus CAU has shown a mean effect size of 0.30 (Weisz et al. 2006), which is lower than the average effect of 0.54 based on comparisons of active treatments with control conditions, most of which were passive or inert (i.e., no treatment, attention control, or waitlist groups) (Weisz et al. 1995). Thus, smaller effect sizes in the present study when compared to other studies that used a no treatment control condition may also be due to the add-on design.

Surprisingly, teachers did report a significant decrease in externalizing behavior problems while parents did not. A possible explanation is that parents in the intervention condition are more motivated than parents in the control condition to report these problems. Indeed, parents learn to observe their child’s behavior and to identify their child’s behavior problems as goals in the parent training (Webster-Stratton 1998). Another possible explanation is that the intervention effects on child behavior are limited to structured settings where children interact with peers and are accompanied by an authoritative person who is not the parent, similar to the intervention setting. In addition, differences between parent-rated outcomes and teacher-rated outcomes are common and may be caused by subtle contextual differences (Grietens et al. 2004). Nonetheless, that effects were based on information from teachers who did not participate in the intervention attests to their robustness.

With regard to clinical change based on T-scores, the intervention significantly increased recovery from (sub)clinical to the normal range, compared to the control group, according to teacher ratings. These findings are in line with the findings on raw scores. However, it also shows that SST in combination with CAU is not more effective than CAU alone in decreasing externalizing behavior from the (sub)clinical to the normal range, according to parent ratings. This might be due to the selection procedure where families were included in the study based on a subclinical level of externalizing behavior in the treatment center or at home, leaving less room for improvement for the children that scored below subclinical levels in one of these settings at pretest. Also, the ‘add on design’ of the study might have reduced the effects with regard to clinical change, as explained earlier in the discussion. However, also including children who show externalizing behavior in one of the two settings is important as this represents the clinical situation where the intervention is performed best. Some of these families were included in the treatment centers due to problems in their family, where parents with low cognitive functioning have reduced capacity to handle their child’s externalizing behavior and MBID, despite the lack of externalizing problems at schools or treatment center settings. In summary, the intervention produced significant reductions in rates of externalizing behavior and significantly changed the percentage of children who were in the subclinical-to-clinical range of these behaviors stronger in the intervention group, compared to the control group according to teacher ratings, but not parent ratings.

As expected, SST was effective in improving positive parenting, extending previous studies in children without MBID that showed improvements in parenting skills as a result of intervention (Lochman and Wells 2002; Webster-Stratton and Hammond 1997). No significant effects, however, were found for negative parenting in this study. A possible explanation is that baseline levels of the Negative Parenting scale were very low in both the intervention and control condition, leaving no room for improvement. The items used in the Negative Parenting scale referred to severe harsh parenting such as ‘You hit your child with a belt when he/she does something wrong’ and ‘I shake my child when we have a fight’. Perhaps, parents that slap their child as a punishment might not have recognized themselves in the items used in our questionnaire, while they would have with less severe items.

Regarding the parent–child relationship, SST combined with CAU was more effective in improving the parent–child relationship than CAU alone. It seems especially important for children with MBID to target the parent–child relationship. Given the impairments of children with MBID, the parent–child relationship may be particularly important as a buffer against inadequate parental responses to disruptive behavior. The parent–child relationship is associated with externalizing child behavior in children with MBID (Schuiringa et al. 2015), and therefore improving the parent–child relationship might lead to improvements on externalizing child behavior.

Unfortunately, effects of the intervention on social cognition were complicated by moderation through type of care. Children who received outpatient treatment benefited from SST, as their aggressive social cognitions marginally reduced more at post-test, compared to the control group. On the other hand, SST had a negative effect on children’s aggressive social cognitions in day care treatment centers, as these children’s aggressive social cognitions increased at post-test. Part of the children in the intervention group thus had more aggressive social cognitions after participating in the intervention, than children in the control group, although these aggressive social cognitions were not associated with externalizing behavior problems. This increase of aggressive cognitions contradicts earlier findings from intervention studies, which showed that aggressive behavior could be treated or prevented by improving the social information processing of children with average intelligence (e.g., Lochman and Wells 2002). This may have been the result of deviancy training as children in day care, in contrast to those receiving the intervention in an outpatient setting, interact more intensively with each other both prior and after the intervention sessions (Dishion and Dodge 2005; Dishion et al. 1999). A comparison between group delivery and individual delivery of the Coping Power program indicates a stronger decrease of externalizing behavior in the individual delivery group, especially for children with low initial levels of inhibitory control (Lochman et al. 2015).

Importantly, it should be noted that clinical workers providing Standing Strong Together were not specifically trained to prevent deviancy training. In addition, children with more severe externalizing problem behavior are treated in day care groups, while children with less severe behavior problems may receive outpatient treatment; thus the peer context between these settings is different. In a severe problem behavior context aggressive problem solving might be perceived to be more ‘effective’ than pro-social problem solving. However, fortunately, we did not find deviancy effects on aggressive behavior. In sum, potential iatrogenic effects on aggressive social cognitions in specific (peer) contexts are a serious concern and deserve much more attention than was given in the present intervention.

Partly in contrast to expectations, no moderation effect of IQ on the intervention effects was found in the current study. Apparently, the child section of the intervention program was sufficiently adapted to the cognitive abilities of children with MBID to accommodate children with IQ’s in the whole range from 55 to 85 to the same extent. Presumably, if cognitive based interventions are adapted in such a way that it well fits the cognitive abilities and needs of children with MBID, the effectiveness of the intervention program will not differ between children with low or relatively higher IQ *within* the IQ range of children with MBID (IQ 55–85). This may also be true for the parent section of the intervention program. We know from the literature that some parents of children with MBID may have low levels of cognitive functioning themselves (De Beer 2012; Van Nieuwenhuijzen et al. 2006). The parent section of the intervention program was developed in such a way that it would fit different cognitive abilities, including low cognitive functioning. In addition, the professional training staff providing SST was experienced and educated in working with parents and children with low cognitive functioning.

The present study concerned the overall effects of a combined parent and child intervention. Given debate about the feasibility of cognitive intervention techniques with children with MBID, it would be interesting to know to what extent the effects we found were due to either the child or the parent component of the intervention. The present study design does not allow us to tease these effects apart, and was not designed to do so. It may be tempting to interpret effects on parenting as due to the parent component and to ascribe effects on social cognitions to the child component, but developmental theory suggests that such a one-on-one interpretation is not warranted. Behavior problems are maintained by continuous transactions between parent and child behaviors and cognitions (e.g., Dodge et al. 2006), so it seems likely that changes induced by the child component may have affected parent behaviors and vice versa. Having established that that externalizing behavior problems of children with MBID can be influenced to some extent, future experimental studies may examine which (parts of) components are responsible for specific effects in specific families.

## Limitations

Results of the study have to be interpreted with care, due to several limitations that might have affected the results. First, it is unclear whether results are generalizable, as (1) we do not have a full view on the representativeness of the study results. The first part of the selection procedure of participants was not fully transparent, as clinicians selected and contacted families based on our inclusion criteria to warrant privacy before consent, (2) due to the dense population in the Netherlands and organization of treatment centers, day treatment can be organized close to families’ homes. This may not be feasible in other countries, and (3) CAU in the Netherlands may be different compared to CAU in other countries.

Second, follow up data are lacking, therefore no conclusions can be drawn on the persistence of the intervention effects. Third, in the present study we relied on rating scales rather than observational measures of externalizing behavior problems and parenting behavior. Fourth, intervention effects were based on child, parent, and teacher reports. However, informants were not blind to conditions.

Nonetheless, this is the first randomized controlled trial, including multi informants, with a large sample, to examine the effectiveness of a combined parent and child intervention specifically aiming at children with MBID and externalizing behavior problems.

## Implications for Research and Clinical Practice

This study demonstrated that it is feasible to effectively conduct a multicomponent treatment with children with MBID and their parents, provided that the cognitive based elements of the intervention program are adapted to their lower cognitive functioning. In the future, it would be interesting to distinguish between effectiveness of the child and parent part of the intervention. In addition, attention should be paid to testing whether changes in parenting behavior and the parent–child relationship caused the reduction in externalizing behavior problems. Future research with longitudinal data as well as more time points is needed to test for mediational effects and effects of specific elements of the intervention.

We find it important to continue to strive to increase the effectiveness of SST in the future. One possibility to increase effectiveness might be to intensify training to SST trainers, by improving their therapeutic skills (Lochman et al. 2009). The present study shows that delivering a multicomponent intervention program which combines a group based parent training and a group based cognitive behavioral therapy for children with MBID and externalizing behavior problems is feasible, and has the potential to reduce children’s externalizing behavior problems and improve both parenting behavior and the parent–child relationship.
